# The Association Between Tongue Pressure and Body Mass Index, Lower-Limb Muscle Strength, and Muscle Mass in Community-Dwelling Older Adults

**DOI:** 10.7759/cureus.109713

**Published:** 2026-05-26

**Authors:** Tomoko Yamashita, Kazuhiko Yamashita, Hiroshi Seno, Hizuru Miyamoto, Yuka Sumita, Shuichi Ino

**Affiliations:** 1 Department of Clinical Engineering, Tohto University, Faculty of Human Care at Makuhari, Chiba, JPN; 2 Department of Core Informatics, Osaka Metropolitan University, Graduate School of Informatics, Osaka, JPN; 3 Department of Applied Informatics, Chiba Institute of Technology, Faculty of Innovative Information Science, Chiba, JPN; 4 Research Center for Advanced Science and Technology, The University of Tokyo, Tokyo, JPN; 5 Dental Clinic, Dentistry and Oral Surgery of Happy Town, Saitama, JPN; 6 Department of Partial and Complete Denture, The Nippon Dental University, School of Life Dentistry at Tokyo, Tokyo, JPN; 7 Graduate School of Engineering, The University of Osaka, Osaka, JPN

**Keywords:** community-dwelling older adults, lower-limb muscle strength, muscle mass, oral hypofunction, tongue pressure

## Abstract

Background and objective

Tongue pressure (TP) is an objective and quantitative measure of tongue function, and low TP is recognized as a component of oral hypofunction. In this cross-sectional study, we aimed to estimate the proportion of community-dwelling older adults who met the existing cutoff for low TP and to examine associations between TP, lower-limb muscle strength, BMI, and muscle mass.

Methods

This study included 447 independently ambulatory adults aged ≥ 70 years. TP was measured with a standardized tongue-pressure device. Lower-limb muscle strength was assessed using toe gap and knee gap force, and muscle mass was evaluated using a body composition analyzer. Participants were stratified by fall-risk classification (based on lower-limb muscle strength) and muscle mass relative to the sample mean. Group comparisons were conducted using analysis of variance and independent-samples t-tests.

Results

More than half of the participants (53.0%) had low TP (<30 kPa). Participants at fall risk based on low toe or knee gap force had significantly lower TP than those not at risk. TP was higher in participants with muscle mass above the mean, and this difference was significant in males. Although lower BMI was associated with low TP, TP differences were more consistent across muscle mass and lower-limb strength classifications.

Conclusions

Low TP was associated with decreased muscle mass and lower-limb muscle strength in community-dwelling older adults. These findings suggest that TP may be related to systemic muscle function rather than oral function alone.

## Introduction

Oral hypofunction is defined based on the assessment of seven indicators, including tongue pressure (TP) [1]. Similar assessment frameworks have been adopted in several countries [2,3]. TP is an objective, quantitative measure of tongue function [4], and low TP has reportedly been associated with impaired swallowing [5], which can lead to decreased food intake and subsequent malnutrition [6]. Furthermore, low TP in community-dwelling older adults has been suggested to be a potential predictor of health risks, including physical frailty [7].

Oral hypofunction conceptually overlaps with oral frailty and is used as a clinical diagnostic framework in Japan; however, its reported prevalence varies widely, ranging from 19.3% to 66.7% [8,9]. Oral hypofunction is reportedly associated with aspiration pneumonia and physical decline, potentially mediated by impaired swallowing and deterioration of the oral environment [10]. Notably, some older adults who meet the diagnostic criteria for oral hypofunction do not subjectively perceive difficulty swallowing [11]. To promote prevention, it is important to clarify the proportion of individuals who exhibit oral hypofunction, that is, oral frailty, among community-dwelling older adults in good health with preserved walking ability.

A decline in oral function is reportedly associated with both reduced oral muscle strength and systemic muscle weakness [12]. Previous studies investigating the relationship between oral function and muscle strength have shown a correlation between swallowing function and mid-upper-arm circumference in hospitalized patients [13]. In contrast, other studies have reported no association between TP and lower-limb muscle strength [14]. However, these studies were limited by their small sample sizes and the inclusion of patients with underlying conditions, such as cancer or dementia. Conversely, large-scale studies conducted in broader populations have reported a substantial association between TP and handgrip strength [15].

In addition to a decline in oral function, reduced lower-limb muscle strength may also contribute to physical decline and decreased appetite in older adults [16]. It has been hypothesized that declines in oral function and lower-limb function occur concurrently. In this context, reduced lower-limb function has been discussed in terms of “foot frailty,” which encompasses reduced toe and lower-limb muscle strength and associated foot conditions. However, few large-scale studies have examined the relationships among TP, lower-limb muscle strength, and muscle mass in physically active, community-dwelling older adults. Although associations between oral function and systemic muscle decline have been reported in frail or hospitalized populations, it remains unclear whether similar relationships are already present in independently ambulatory, community-dwelling older adults. Clarifying these associations may contribute to the early identification of physical frailty before overt disability develops.

Therefore, in this cross-sectional study, we aimed to determine the proportion of community-dwelling older adults who met the existing cutoff values for low TP and to examine the associations between TP and lower-limb muscle strength, BMI, and muscle mass.

## Materials and methods

Participants and measurements

This study was conducted in Shiki City, Saitama Prefecture, Japan. The participants were 447 community-dwelling older adults aged ≥70 years (mean age: 79.3 ± 3.9 years; range: 72-91 years). The participants included 208 males (mean age: 79.8 ± 4.2 years, BMI: 23.3 ± 2.7 kg/m^2^) and 239 females (mean age: 79.0 ± 3.5 years, BMI: 22.3 ± 3.7 kg/m^2^). The baseline characteristics of the study participants are presented in Table 1.

**Table 1:** Baseline characteristics of the participants SD: standard deviation

Variables	Male	Female	Total
Number of participants	208	239	447
Age, years, mean (SD)	79.8 (4.2)	79.0 (3.5)	79.4 (3.9)
BMI, kg/m^2^, mean (SD)	23.3 (2.7)	22.3 (3.7)	22.7 (2.9)
Muscle mass, kg, mean (SD)	46.9 (4.6)	33.4 (2.7)	39.7 (7.7)
TP, kPa, mean (SD)	29.6 (7.9)	28.4 (7.8)	28.9 (7.9)
Toe gap force_Right, kgf, mean (SD)	4.1 (1.5)	3.3 (1.2)	3.7 (1.4)
Toe gap force_Left, kgf, mean (SD)	3.6 (1.5)	2.9 (1.2)	3.3 (1.4)
Knee gap force, kgf, mean (SD)	18.7 (5)	14.6 (3.9)	16.5 (4.9)

All participants were recruited through a public announcement issued by the local municipality and were able to walk independently without assistance. Participants who were unable to complete the measurements or had severe cognitive impairment or acute illness at the time of assessment were excluded. This study was conducted in accordance with the principles of the Declaration of Helsinki and was approved by the Ethics Committee of Tohto University (approval number: R0701).

**Figure 1:**
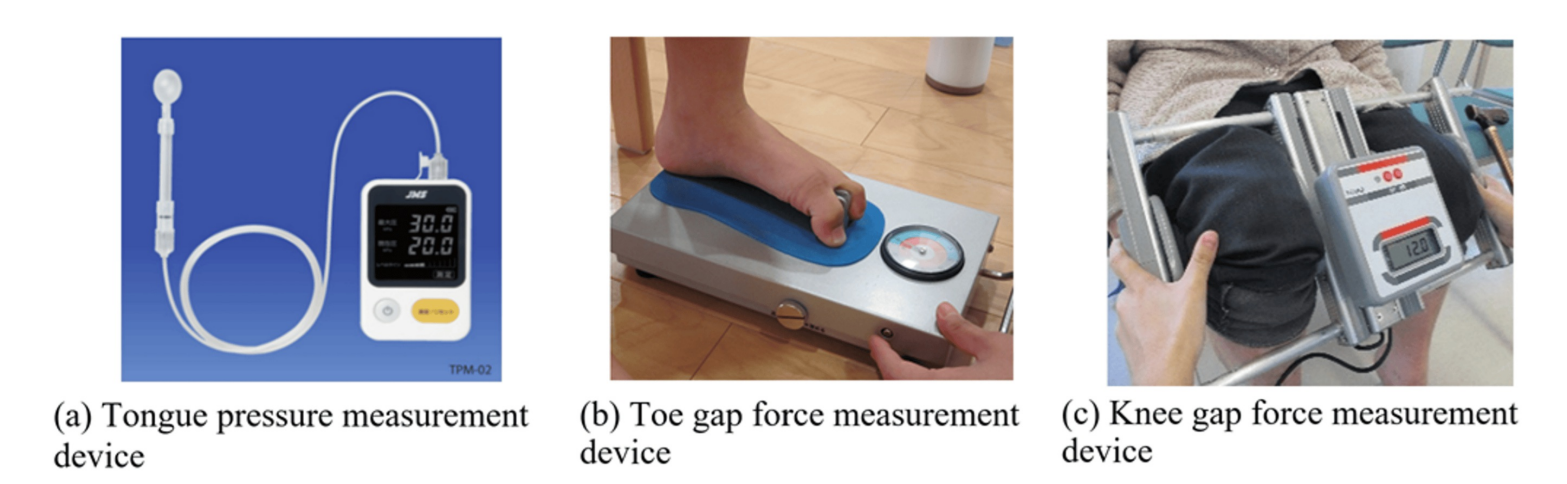
Measurement devices

Measurements included TP (Figure 1a), toe gap force (Figure 1b), knee gap force (Figure 1c), BMI, and muscle mass. The TP was measured using a TP measurement device (TPM-02E, JMS Co., Ltd., Tokyo, Japan); measurements were performed three times, and the maximum value was recorded for analysis. All measurement devices were calibrated and prepared according to the manufacturers’ recommendations before data collection. Based on previously proposed diagnostic criteria for oral hypofunction, a TP value <30 kPa was defined as low TP [1]. This cutoff is widely used clinically in Japan for screening oral hypofunction in older adults.

BMI was categorized as follows: underweight (<18.5 kg/m²), normal weight (18.5 to <25.0 kg/m²), and overweight/obese (≥25.0 kg/m²). Participants with a BMI <18.5 kg/m² were classified as underweight, which, in older adults, may be regarded as indicative of increased risk for sarcopenia.

Toe gap force (Nisshin Sangyo Co., Ltd., Saitama, Japan) was measured in a seated position by instructing the participants to grasp the measurement bar with the great and second toes, thereby assessing lower-limb muscle strength [17]. The left and right feet were tested twice, and the highest value was used for analysis. The cutoff values for fall risk based on toe gap force for males and females were ≤3.0 and ≤2.5 kgf, respectively [17].

Knee gap force (Nisshin Sangyo Co., Ltd.) was measured with the participant in a seated position by placing the device between the knees and instructing the participants to press inward, thereby assessing hip adductor muscle strength [18]. Measurements were performed twice, and the highest value was used for analysis. The cutoff values for fall risk based on knee gap force for males and females were ≤10 and ≤8 kgf, respectively [18].

Muscle mass was assessed using a bioelectrical impedance analysis (BIA)-based body composition analyzer (UC-421BLE, A&D Co., Ltd., Tokyo, Japan). Measurements were performed with participants standing barefoot in an upright position according to the manufacturer’s instructions. The device estimated skeletal muscle mass based on bioelectrical impedance and anthropometric parameters. Participants were instructed to avoid excessive physical activity immediately before measurement to minimize measurement variability.

Statistical analysis

Statistical analyses were performed using SPSS Statistics (version 30; IBM Corp., Armonk, NY). Descriptive statistics are presented as means (standard deviations (SD). Analysis of variance (ANOVA) was used to compare TP across the BMI categories. Comparisons of TP according to lower-limb muscle strength were conducted using independent-sample t-tests comparing fall-risk vs. non-fall-risk groups separately for toe and knee gap force.

Data normality was assessed using the Shapiro-Wilk test. Parametric tests were applied when the assumption of normality was satisfied; otherwise, non-parametric alternatives (Mann-Whitney U test) were used. P-values < 0.05 were considered statistically significant. Homogeneity of variances was evaluated using Levene’s test.

## Results

**Figure 2:**
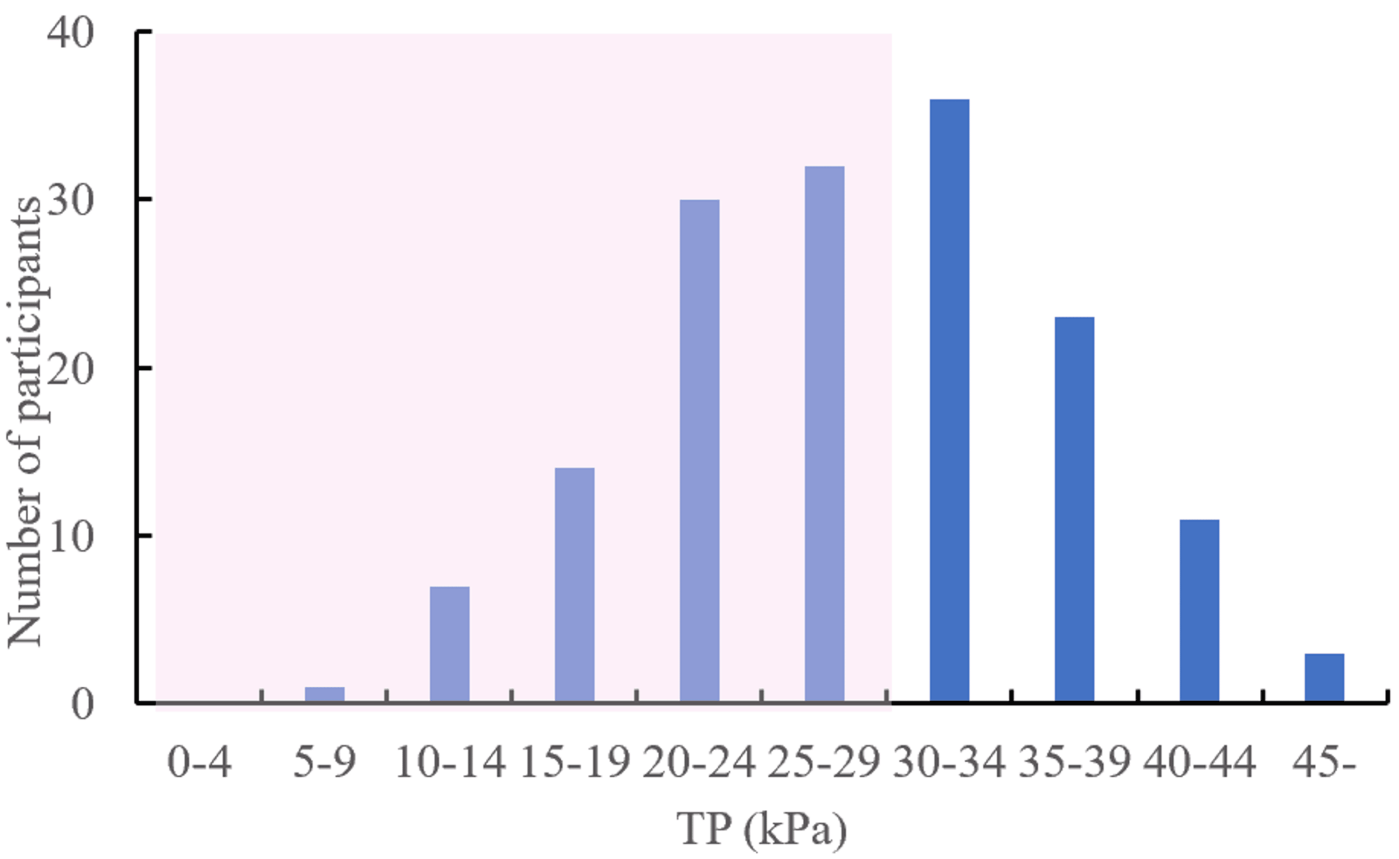
Distribution of TP in community-dwelling older adults The pink shaded area indicates participants with low tongue pressure (< 30 kPa) TP: tongue pressure

**Figure 3:**
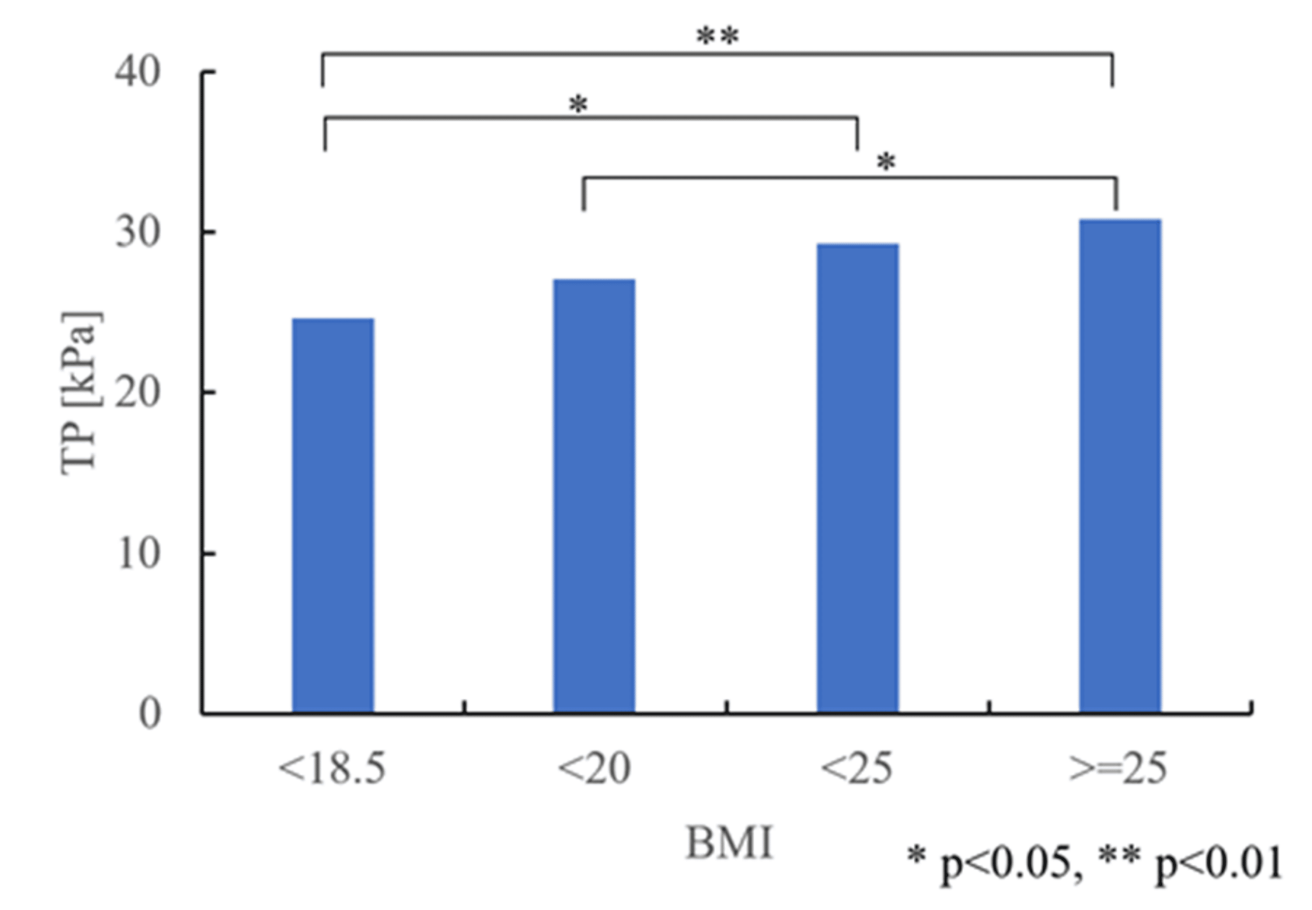
Comparison of TP across BMI categories TP: tongue pressure; BMI: body mass index

Figure 2 shows the histogram of TP. Overall, 53.0% of the participants were classified as having TP values below 30 kPa. Figure 3 shows TP stratified by BMI category, with a progressive decrease in TP observed with decreasing BMI. In particular, the underweight group (BMI < 18.5) exhibited significantly lower TP than did the normal-weight and overweight groups (p = 0.01 and < 0.001, respectively). TP was 17.0% and 10.6% lower in the underweight and BMI <20 groups, respectively, compared with the normal-weight group.

**Figure 4:**
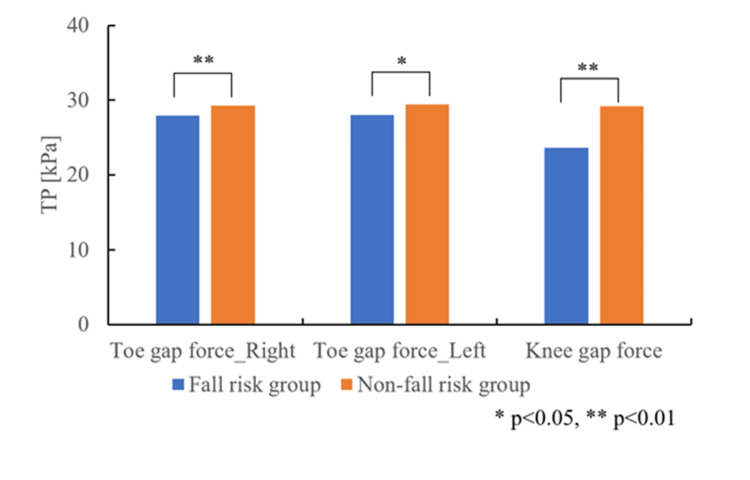
Association between TP and fall risk based on lower limb muscle strength TP: tongue pressure

Figure 4 shows TP values according to fall risk classification based on lower-limb muscle strength. Compared with the non-fall-risk group, the fall-risk group defined by toe gap force exhibited lower TP values (p < 0.05). In addition, participants classified as being at fall risk owing to low knee gap force (hip adductor muscle strength) demonstrated TP values that were approximately 20% lower than those of participants not at risk (p < 0.01).

Table 2 shows a comparison of measured variables between the groups stratified by the sex-specific mean value of muscle mass, defined as below or above the mean value. TP was higher in the group with muscle mass above the mean than in the group with muscle mass below the mean, with 1.14- and 1.04-fold differences in males and females, respectively. A statistically significant difference was observed only among males (male: p < 0.001, female: p = 0.12).

**Table 2:** Comparison of measured variables between groups stratified by muscle mass (below vs. above the mean) in males and females Groups were defined based on the mean value of muscle mass. P-values were calculated using t-tests SD: standard deviation; CI: confidence interval

	Male					Female				
	Below mean muscle mass, mean (SD)	Above mean muscle mass, mean (SD)	t-value	p-value	95% CI	Below mean muscle mass, mean (SD)	Above mean muscle mass, mean (SD)	t-value	p-value	95% CI
Age, years	80.4 (4.5)	79.0 (3.8)	2.48	0.01	0.29 - 2.56	79.0 (3.5)	78.8 (3.6)	0.45	0.33	-0.7 - 1.12
BMI, kg/m^2^	21.8 (2.3)	24.6 (2.4)	-8.49	<0.001	-3.42 - -2.13	20.8 (2.5)	23.9 (2.8)	-9.0	< 0.001	-3.76 - -2.41
TP, kPa	27.6 (7.3)	31.5 (8.0)	-3.68	<0.001	-6.01 - -1.81	27.9 (8.0)	29.1 (7.5)	-1.20	0.12	-3.2 - 0.78
Muscle mass, kg	43.2 (3.0)	50.4 (2.6)	-18.63	<0.001	-7.99 - -6.46	31.3 (1.6)	35.7 (1.6)	-21.01	< 0.001	-4.79 - -3.97
Toe gap force_Right, kgf	4.0 (1.5)	4.2 (1.4)	-0.82	0.21	-0.58 - 0.24	3.2 (1.2)	3.4 (1.1)	-1.30	0.097	-0.49 - 0.1
Toe gap force_Left, kgf	3.5 (1.5)	3.7 (1.4)	-0.93	0.18	-0.6 - 0.21	2.9 (1.3)	3.0 (1.0)	-0.38	0.35	-0.36 - 0.25
Knee gap force, kgf	17.5 (4.9)	19.8 (4.8)	-3.53	<0.001	-3.69 - -1.05	13.9 (3.7)	15.4 (4.0)	-3.09	0.001	-2.51 - -0.55

## Discussion

This study focused on TP and examined its association with BMI and lower-limb muscle strength in relatively healthy, independently ambulatory, community-dwelling older adults. According to the current diagnostic criteria for oral hypofunction in Japan, low TP is defined as a maximum TP of less than 30 kPa [1]. In contrast, previous studies have proposed lower threshold values for impaired tongue function, specifically 24.3 kPa for males and 23.7 kPa for females [19]. In this study, a cutoff value of 30 kPa was adopted in accordance with these criteria. Consequently, more than half of the participants were classified as having low TP. When the lower, sex-specific thresholds described above (24.3 kPa for males and 23.7 kPa for females) were applied, the proportion classified as having low TP was 31.1%. The first quartile values of TP observed in this study were 23.9 kPa for males and 22.9 kPa for females, slightly lower than those reported in previous studies. This difference may be attributable to the advanced age of the participants in this study, all of whom were aged ≥ 70 years.

Oral hypofunction involves multiple indicators, including poor oral hygiene, oral dryness, reduced occlusal force, impaired masticatory function, and impaired swallowing function [1]. Therefore, the appropriateness of a fixed cutoff value of 30 kPa for TP warrants further consideration. Cutoff values derived from sufficiently large age- and sex-stratified samples may be more suitable for accurately identifying clinically meaningful impairment [19]. In this study, more than 50% of the participants met this criterion. Therefore, the high prevalence observed in this relatively healthy cohort suggests that the currently used cutoff value may classify a substantial proportion of community-dwelling older adults as having low TP, and that this classification should be interpreted with caution in this context.

Low TP has been reported to be associated with sarcopenia [20], which is characterized by a generalized decline in skeletal muscle mass and strength, and is suspected to induce functional deterioration of the oral musculature, including the masticatory and tongue muscles [21,22]. Accordingly, low body weight is often used as a practical, readily identifiable indicator of sarcopenia [23,24]. Although previous studies have suggested that low body weight may influence oral function, the extent to which it is directly associated with low TP remains unclear [25,26].

In this study, the association between BMI and TP was examined to determine the relationship between low body weight and tongue function. Participants in the underweight group (BMI < 18.5) exhibited approximately 17.0% lower TP than those in the normal-weight group. TP was 10.6% lower among individuals with a BMI < 20, indicating that TP decreased as BMI decreased. In older adults, a low BMI (e.g., < 20) is associated with an increased risk of sarcopenia [23]; therefore, systemic muscle weakness may be associated with oral functional decline. Unlike previous studies that primarily focused on hospitalized or frail populations, our findings demonstrated an association between TP and lower-limb muscle strength, even among physically active, community-dwelling older adults. Specifically, participants classified as being at a higher risk of falls owing to reduced lower-limb muscle strength also exhibited lower TP. These findings suggest that declines in oral function and lower-limb muscle strength may occur concurrently. A previous study examined handgrip strength and knee extension strength and reported that decreased tongue pressure was associated with handgrip strength in men and knee extension strength in women. Although the muscle groups differed between the upper and lower extremities, these findings were consistent with the present results [27].

In this study, we focused on not only muscle strength (force-generating capacity) but also muscle mass. Stratified analyses based on mean muscle mass and lower-limb muscle strength were also conducted. A lower TP was observed in participants with lower muscle mass. Although this difference was statistically significant in males, a similar decreasing trend, although not significant, was observed in females. Previous studies have reported that age-related hormonal changes, particularly declines in estrogen levels, influence skeletal muscle mass and function in females [28]. Such sex-specific factors may be one possible explanation for why the difference in TP between the muscle-mass groups did not reach statistical significance in females in this study. Moreover, when participants were stratified by muscle mass, BMI differed significantly between the groups in both males and females, with a lower BMI observed in those with lower muscle mass.

These patterns are consistent with the association between lower BMI and lower TP observed in this study. Taken together, lower muscle mass and reduced lower-limb strength co-occurred with low TP in this cohort, and TP may help identify individuals with broader muscle function decline. In addition, TP may serve as a useful indicator of early physical frailty in older adults. One possible explanation is that generalized age-related skeletal muscle decline may affect not only appendicular muscles but also the tongue musculature. In addition, reduced nutritional intake associated with impaired oral function may further contribute to systemic muscle loss.

Previous studies have reported that foot frailty, characterized by reduced lower-limb muscle strength and foot-related conditions such as toenail and foot deformities, is associated with an increased risk of falls and a subsequent decline in physical activity [29,30]. From the perspective of reduced activity levels, declines in oral function and foot frailty may be interrelated. Accordingly, the coexistence of impaired lower-limb muscle strength and decreased oral function may further increase the risk of functional dependence in older adults.

This study has several limitations. First, because of the cross-sectional design, causal relationships between tongue pressure and systemic muscle function cannot be established. Second, although the participants were independently ambulatory older adults, potential confounding factors such as nutritional status, physical activity level, comorbidities, and medication use were not fully adjusted for. Third, muscle mass was assessed using a BIA-based device rather than dual-energy X-ray absorptiometry. Therefore, further longitudinal studies are needed to clarify the mechanisms underlying the observed associations.

## Conclusions

The findings of this study demonstrate that low TP, an indicator of impaired oral function, is associated with lower BMI, lower-limb muscle strength, and muscle mass in community-dwelling older adults. These factors are closely related to declines in physical activity and fitness and may contribute to deterioration in activities of daily living among older adults. If TP measurement can be implemented as a simple, quantitative assessment tool, it may facilitate the early identification of individuals at risk of physical frailty and support preventive strategies aimed at maintaining functional independence among older adults.
